# Human Polyomavirus 9 Infection in Kidney Transplant Patients

**DOI:** 10.3201/eid2006.140055

**Published:** 2014-06

**Authors:** Els van der Meijden, Herman F. Wunderink, Caroline S. van der Blij-de Brouwer, Hans L. Zaaijer, Joris I. Rotmans, Jan Nico Bouwes Bavinck, Mariet C.W. Feltkamp

**Affiliations:** Leiden University Medical Center, Leiden, the Netherlands (E. van der Meijden, H.F. Wunderink, C.S. van der Blij-de Brouwer, J.I. Rotmans, J.N. Bouwes Bavinck, M.C.W. Feltkamp);; Sanquin Blood Supply Foundation, Amsterdam, the Netherlands (H.L. Zaaijer)

**Keywords:** polyomavirus, HPyV9, BKPyV, immunocompromised host, kidney transplantation, kidney transplant, viruses, BK polyomavirus, transplant, immunosuppression, kidney-pancreas transplant

## Abstract

Several human polyomaviruses of unknown prevalence and pathogenicity have been identified, including human polyomavirus 9 (HPyV9). To determine rates of HPyV9 infection among immunosuppressed patients, we screened serum samples from 101 kidney transplant patients in the Netherlands for HPyV9 DNA and seroreactivity. A total of 21 patients had positive results for HPyV9 DNA; positivity rates peaked at 3 months after transplantation, but the highest viral loads were measured just after transplantation. During 18 months of follow-up, HPyV9 seroprevalence increased from 33% to 46% among transplant patients; seroprevalence remained stable at ≈30% in a control group of healthy blood donors in whom no HPyV9 DNA was detected. Further analysis revealed an association between detection of HPyV9 and detection of BK polyomavirus but not of cytomegalovirus. Our data indicate that HPyV9 infection is frequent in kidney transplant patients, but the nature of infection—endogenous or donor-derived—and pathogenic potential of this virus remain unknown.

## Abstract 2

This virus is frequently found within the first year after transplantation and in association with BK polyomavirus infection.

The *Polyomaviridae* constitute a family of small DNA viruses that infect a variety of hosts. BK polyomavirus (BKPyV) and JC polyomavirus (JCPyV), discovered in 1971 (*1*,*2*), are well-known examples of human polyomaviruses (HPyVs) that cause severe disease in immunocompromised patients. Serologic data have revealed that most polyomaviruses are ubiquitous (*3*–*6*). In case of JCPyV and BKPyV, primary infection occurs early in life, without apparent symptoms, and persists throughout life as latent infection in the kidneys, accompanied by occasional virus shedding in urine (*7*). When immunity is decreased, these viruses can reactivate with detectable viremia and manifestation of disease, which poses a threat to, among others, patients who receive solid-organ transplants. For kidney transplant patients, BKPyV infection is considered the most common viral complication and causes nephropathy and graft loss in 1%–10% of cases if left untreated (*8*). It is not known what determines the severity of BKPyV infection and whether co-infection is involved in the pathogenesis. 

Since 2007, at least 10 novel HPyVs have been discovered (*9*–*20*); of these, Merkel cell polyomavirus (MCPyV) and trichodysplasia spinulosa–associated polyomavirus (TSPyV) have been shown to be associated with disease (*11*,*19*,*21*,*22*). Human polyomavirus 9 (HPyV9), so far without a disease association, was identified in 2011 from a serum sample from a kidney transplant patient (*17*). Overall seroprevalence of HPyV9 has been determined to be 25% to 50% (*23*–*26*).

Because HPyV9 was originally isolated from a kidney transplant recipient (*17*), we aimed to systematically study the presence of HPyV9 infection in kidney transplant patients and investigate a possible association with the known nephropathogenic BKPyV. We analyzed a cohort of 101 transplant patients who received either a kidney transplant or a simultaneous kidney–pancreas transplant for the appearance of markers for HPyV9 infection during the 18 months after transplantation. We assessed the presence of HPyV9 DNA and IgG seroresponses in serum samples. The HPyV9 findings in the transplant cohort were compared with those obtained for an age- and sex-matched cohort of healthy blood donors. Co-infection with BKPyV was investigated by comparing observed HPyV9 and BKPyV viremia levels in the transplant cohort. For comparative purposes, we also tested for cytomegalovirus (CMV), which, like polyomaviruses, frequently reactivates during immunosuppressive drug use after transplantation.

## Materials and Methods

### Study Population

The cohort study consisted of 101 patients who received kidney (n = 83) or kidney–pancreas (n = 18) transplants during 2002–2004 at Leiden University Medical Center (LUMC), Leiden, the Netherlands (Table 1). This study population is part of a larger prospective European multicenter study designed to investigate the role of human papillomavirus infection in the development of skin cancer in solid-organ transplant patients (*27*). The study adhered to the Declaration of Helsinki Principles, and the medical ethical committee of the LUMC approved of the study design (Medical Ethical Committee no. P02.111). Participants gave written informed consent. 

**Table 1:** Characteristics of patients and controls for study of human polyomavirus 9 prevalence among kidney transplant patients, the Netherlands*

Characteristic	Transplant patient type				Blood donors
	All	Kidney	Kidney–pancreas	p value	
Patients and controls	101	83 (82)	18 (18)		87
Mean age, y (range)	47 (21–74)	48 (21–74)	43 (30–54)	0.129†	52 (29–68)
Sex					
F	34 (34)	27 (33)	7 (39)	0.605‡	31 (36)
M	67 (66)	56 (67)	11(61)		56 (64)

*Values are no. (%) except as indicated. †Comparison of kidney and kidney–pancreas patient groups; Student *t* test. ‡Comparison of kidney and kidney–pancreas patient groups; χ^2^ test.

All patients received induction with interleukin-2 receptor blocker daclizumab (100 mg/d) on the day of transplantation and 10 days after transplantation or basiliximab (40 mg at days 0 and 4), followed by triple therapy with prednisone, tacrolimus, or cyclosporine and mycophenolate mofetil. For kidney transplant patients, the dose of the calcineurin inhibitor (tacrolimus or cyclosporine) was tapered at 6 weeks after transplantation, whereas for kidney–pancreas transplant patients, the calcineurin inhibitor was reduced at 3 months after transplantation.

The time points of serum sample collection and the number of samples collected per time point are summarized in Table 2 and shown in relation to the date of transplantation. The baseline samples were obtained in the days immediately after transplantation (time point 0, T0). To collect the subsequent samples, patients were asked to visit the LUMC outpatient clinic for follow-up sample collection at the preferred time points of 3, 6, 9, 12, and 18 months after transplantation (T3–T18). A total of 58 patients provided a sample at all 6 time points: 31, 6, 4, and 2 patients provided 5, 4, 3, or 1 samples, respectively.

**Table 2:** Detection of human polyomavirus 9 DNA and viral loads in kidney transplant patients and blood donors, the Netherlands*

Population	Mean time after transplantation or first sample collection, mo (range)	No. samples	No. (%) HPyV9 DNA positive	Mean viral load, copies/mL (range)
Transplantation patients		101	21 (20.8)†	157 (25–530)‡
Transplant type				
Kidney		83	17 (20.5)†	135 (25–530)‡
Kidney and pancreas		18	4 (22.2)†	250 (89–472)‡
No. serum samples		541	27 (5.0)	137 (25–530)
Mo after transplant				
Pretransplant§	–0.3 (–1.4 to 0)	65	0	NA
0	0.4 (0.1–1.2)	99	3 (3.0)	203 (141–265)
3	3.5 (2.3–5.5)	98	7 (7.1)	172 (52–530)
6	6.5 (5.5–9.6)	97	6 (6.2)	141 (25–472)
9	9.6 (7.6–12.6)	80	5 (6.3)	125 (45–213)
12	12.6 (9.3–16.0)	87	4 (4.6)	80 (66–92)
18	18.2 (16.0–21.3)	80	2 (2.5)	51 (38–63)
Blood donors		87	0	NA
No. serum samples		174	0	NA
Mo after first sample collection				
0	0	87	0	NA
12	13.4 (9.9–18.1)	87	0	NA

*NA, not applicable. †Patients HPyV9 positive in the follow-up period after transplant. ‡Mean load of HPyV9 DNA–positive patients based on the first positive sample per patient. §Pretransplant samples were retrieved from the serum sample archive at the Leiden University Medical Center Clinical Microbiology Laboratory (Leiden, the Netherlands).

Stored pretransplantation serum samples, if available, were retrieved and tested for HPyV9 DNA (n = 65; Table 2) and antibodies (n = 45, 40 of which were also included in pretransplantation DNA testing). The average dates of obtaining samples for DNA and antibody testing were 8 and 2 days before transplantation, respectively.

To obtain a healthy control population, we analyzed anonymized samples from 87 random unpaid blood donors (Table 2). For each donor, 2 follow-up serum samples were studied, collected 1 year apart (T0 and T12). The donors were matched for age and sex with the transplant patient study population (Table 1).

### Viral DNA Detection and Quantification

Total nucleic acids were extracted from 200 µL serum by using the MagNA Pure LC Total Nucleic Acid Isolation Kit–High Performance and MagNA Pure LC Instrument (Roche Diagnostics, Indianapolis, IN, USA). To monitor the quality of DNA extraction and potential PCR inhibition, we added low concentrations of phocine herpesvirus (*28*) to the lysis buffer. DNA was eluted in a final volume of 100 μL elution buffer, of which 10 μL was used as input for real-time quantitative PCR (qPCR).

Primers and Taqman probes were designed by using Beacon Designer software (Premier Biosoft, Palo Alto, CA, USA). For HPyV9, we used the following primers and probe, located in the viral protein (VP) 1 gene amplifying a product of 109 nt: sense primer 5′- CCTGTAAGCTCTCTCCTTA-3′, antisense primer 5′- CCTGATAAATTCTGACTTCTTC-3′, and probe FAM-5′- CTTGTTCTCTGGTCTTATGCCTCA-3′-BHQ-1. For BKPyV, we used the following primers and probe, located in the VP1 gene amplifying a product of 90 nt: sense primer 5′-GAAAAGGAGAGAGTGTCCAGGG-3′, antisense primer 5′-GAACTTCTACTCCTCCTTTTATTAGT-3′, and probe FAM-5′-CCAAAAAGCCAAAGGAACCC-3′-BHQ1.

The BKPyV qPCR and phocine herpesvirus PCR were duplexed for DNA quality and potential PCR inhibition monitoring. Furthermore, the BKPyV qPCR was validated to detect BKPyV genotypes I–IV. qPCR reactions were performed in a total volume of 50 μL, containing 25 μL HotStart Taq mastermix (QIAGEN, Hilden, Germany), 0.5 µmol/L of each primer, 0.35 µmol/L BKPyV probe or 0.4 µmol/L HPyV9 probe, and 3.5 mmol/L MgCl_2_. Reactions were performed by using a CFX96 real-time detection system (Bio-Rad, Hercules, CA, USA) with the following cycle conditions: 15 min at 95°C followed by 45 cycles of amplification (30 s at 95°C; 30 s at 60°C for HPyV9 qPCR and 55°C for BKPyV qPCR; 30 s at 72°C). For quantification, a standard of pGEX 5×3 HPyV9 VP1 plasmid (Genscript, Piscataway, NJ, USA) and of a quantified BKPyV-positive urine sample were used. Analytical sensitivity of the HPyV9 and BKPyV qPCRs was ≈10 copies/mL. CMV load was measured as described (*29*) with minor adjustments: 0.5 µmol/L of each primer, 0.2 µmol/L probe, and HotStart Taq mastermix (QIAGEN) was used with the following cycle conditions: 15 min at 95°C, followed by 45 cycles of amplification (5 s at 95°C, 15 s at 63°C, 15 s at 72°C). Analytical sensitivity of the CMV qPCR was ≈100 copies/mL. On each plate, 3 negative controls were included; these controls tested negative in all PCR assays. PCR results with a cycle threshold ≥40 were considered negative.

### HPyV9 DNA Sequencing

HPyV9-positive PCR samples were confirmed by sequencing. PCR products were cloned by using the TOPO TA Cloning Kit (Invitrogen, Carlsbad, CA, USA) according to the manufacturer’s instructions and subsequently sequenced. Sequence reactions were performed by using the BigDye Terminator Kit (Applied Biosystems, Foster City, CA, USA) and analyzed on an ABI Prism 3130 Genetic Analyzer (Applied Biosystems).

### HPyV9 Serologic Testing

To detect IgG seroresponses against the major capsid protein VP1 of HPyV9, we performed an antibody-binding assay using Luminex xMAP technology (*30*), as described (*26*). Briefly, the assay is based on cross-linking of glutathione to casein, which is subsequently coupled to fluorescent polystyrene beads (Bio-Rad). Glutathione S-transferase HPyV9 VP1 fusion protein was affinity purified on the beads. Serum samples were tested in a 1:100 dilution, and VP1-bound antibodies were detected with biotinylated goat anti-human IgG (H+L; Jackson Immuno Research, West Grove, PA, USA), followed by streptavidine-R-phycoerythrin (Invitrogen). Finally, the beads and the phycoerythrin signal were analyzed in a Bio-Plex 100 Analyzer (Bio-Rad), which gave results in median fluorescent intensity (MFI). For background correction, MFI values of glutathione S-transferase alone were subtracted to obtain HPyV9 VP1–specific signals. Quality control was performed on each plate with a serum pool consisting of 4 serum samples that had been analyzed in a 1:4 serial dilution, starting with a dilution of 1:100 up to 1:409,600. Little interplate variance was observed.

### Cutoff Value Determination

The cutoff value of the antibody-binding assay was defined on the basis of a group of healthy children 0.5–2 years of age and determined as described by van der Meijden et al. (*26*). The transplant patients and the healthy blood donors were analyzed in 2 independent antibody-binding assays. HPyV9 cutoff values of 252 MFI and 311 MFI were determined for the transplant patient group and the blood donor group, respectively.

### Statistical Analyses

Differences between groups in terms of HPyV9 DNA or seroprevalence were assessed by using the Pearson χ^2^ or Fisher exact test, as appropriate for population size. Independent Student *t* tests or analyses of variance were used for comparisons of mean values between groups. Occurrence of HPyV9 viremia at transplantation was calculated by using the Kaplan-Meier method, with the time from transplantation to the next detected HPyV9 DNA as the outcome variable for HPyV9-seronegative and -positive patients at baseline (T0). HPyV9 seroconversion at transplantation was calculated by using the Kaplan-Meier method with the time from transplantation to the next seropositive sample as the outcome variable for HPyV9 viremic and nonviremic patients during follow-up. For all tests, 2-tailed p values ≤0.05 were considered significant. The statistical analyses in this study were performed by using SPSS 20 (IBM, Armonk, NY, USA) and Prism 3 statistical software (GraphPad Software Inc., San Diego, CA, USA).

## Results

### HPyV9 Viremia in Kidney Transplant Patients

**Figure 1:**
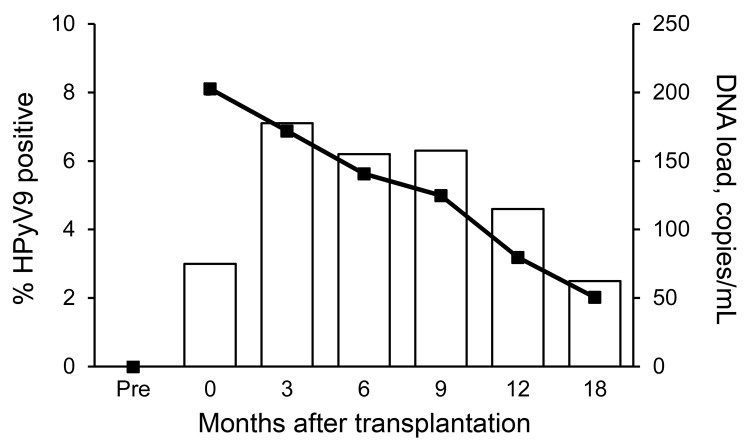
Human polyomavirus 9 (HPyV9) DNA positivity and mean DNA viral load in transplant patients over time, the Netherlands. Bars indicate percentage of HPyV9-positive patients; line indicates DNA load. Time points are shown as described in Table 2. Pre, pretransplant (baseline).

To determine HPyV9 viremia in the transplant patients, we assessed the presence of HPyV9 DNA in the complete sample set. During the 18 months after transplantation, HPyV9 DNA was detected at some point in 21 (20.8%) of the 101 patients (Table 2). No significant difference in the detection of HPyV9 DNA was observed between kidney and kidney–pancreas transplant patients (20.5% vs. 22.2%, respectively). For 3 (3.0%) patients, results were positive for consecutive serum samples; persistent HPyV9 DNA detection throughout the follow-up period was observed for 1 patient. Shortly after transplantation (T0, on average 11 days after transplantation), HPyV9 DNA was detected in 3.0% of patients. Detection of HPyV9 DNA peaked 3 months after transplantation (7.1% positivity) and gradually decreased to 2.5% 18 months after transplantation (Figure 1; Table 2).

### Lack of HPyV9 Viremia before Transplantation and in Controls

To explore the possibility that the observed viremia was not related to the transplant and immunosuppression but to the underlying cause of the kidney disease (e.g., diabetes), we retrieved and analyzed pretransplantation serum samples for 65 (64%) of the 101 transplant patients. In addition, the group of 87 healthy blood donors was analyzed for the presence of HPyV9 DNA. No DNA was detected in either of these sample sets. To confirm the HPyV9-specificity of our PCR findings in the transplant patients, we cloned and sequenced 13 of the 27 HPyV9-positive PCR products, 109 nt in length. The results revealed a complete match with the described HPyV9 DNA sequence in GenBank (accession no. NC_015150) for 12 of 13 samples. In 1 sample, a single nonsynonymous nucleotide mismatch was observed (A→G at position 2403), resulting in an I321V amino acid mutation in the VP1 capsid protein.

### Peak of HPyV9 Viral Load Immediately after Transplantation

The mean HPyV9 DNA load after transplantation was 137 copies/mL (range 25–530 copies/mL). On average, the highest viral loads were observed immediately after transplantation (Figure 1). The kidney–pancreas transplant patients tended to show higher HPyV9 DNA loads than did kidney transplant patients (mean values of 250 and 135 copies/mL, respectively; Table 2), but this difference was not statistically significant (p = 0.123 by Student *t* test).

### HPyV9 Seroreactivity Increase in Transplant Patients but Not in Controls

**Figure 2:**
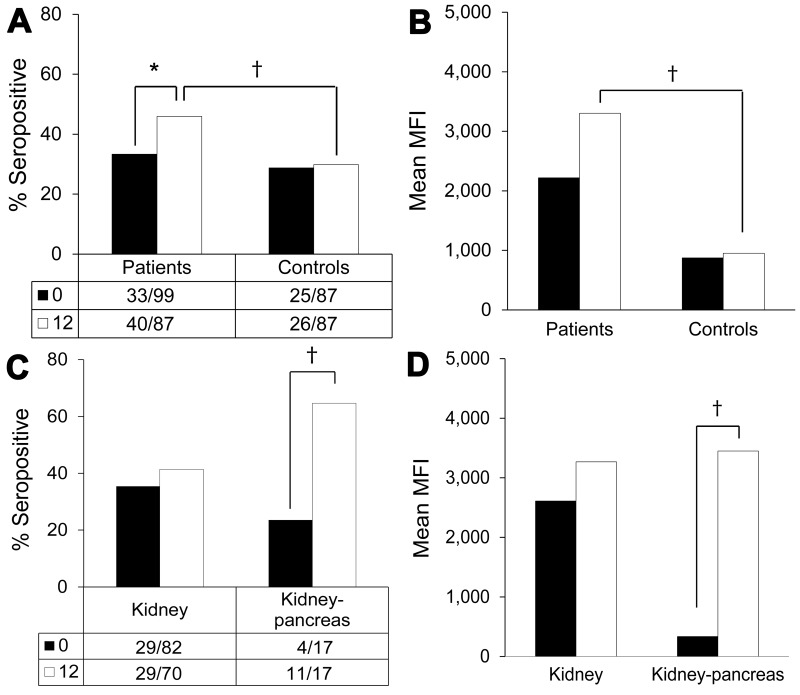
Human polyomavirus 9 (HPyV9) seropositivity and seroreactivity in samples from transplant patients and healthy blood donor controls collected 1 year apart, the Netherlands. Black bars, baseline samples; white bars, follow-up samples (Table 2). Values below bars indicate no. persons positive/total no. tested. A) Seropositivity percentages for transplant patients and controls; B) seroreactivity levels for transplant patients and controls; C) seropositivity percentages for kidney transplant and kidney–pancreas transplant patients; D) seroreactivity levels for kidney transplant and kidney–pancreas transplant patients. MFI, median fluorescent intensity. *Borderline significant (0.05<p<0.100); †significant (p<0.05).

HPyV9 seroresponses were analyzed for the complete sample set. At baseline, just after transplantation (T0), 33% of transplant patients were HPyV9 seropositive. This percentage corresponds to the percentages that we measured in healthy blood donors (29%) and in 45 pretransplantation serum samples (31%). However, at 1 year after transplantation (T12), the seropositivity rate for transplant patients rose to 46%. This percentage differed significantly from the rate measured for blood donors, which remained stable at ≈30% during 1 year of follow-up (p = 0.029 by χ^2^ test) (Figure 2, panel A). In total, 15 (15%) of 101 transplant patients seroconverted during follow-up (Technical Appendix Figure 1); these patients represent 23% (15/66) of the patients who were seronegative at baseline. 

The intensity of measured HPyV9 serologic responses also increased after transplantation, whereas HPyV9 seroreactivity in blood donors was lower at baseline and remained low within a comparable follow-up period of 1 year (Figure 2, panel B). Mean HPyV9 seroreactivity in the 1-year follow-up samples was significantly higher for the transplant group than for blood donors (p = 0.008 by Student *t* test). Further analysis of the transplant population revealed that kidney–pancreas transplant patients in particular were responsible for the observed increase in HPyV9 seroreactivity after transplantation (Figure 2, panels C, D). Kidney–pancreas transplant patients had lower mean seroreactivity at baseline than did kidney transplant patients (Figure 2, panel D); the relative increase of seroreactivity in kidney–pancreas transplant patients was confirmed by analyzing the complete dataset with a mixed model analysis (p = 0.003; data not shown).

### No Correlation between HPyV9 Viremia and Seroreactivity

**Figure 3:**
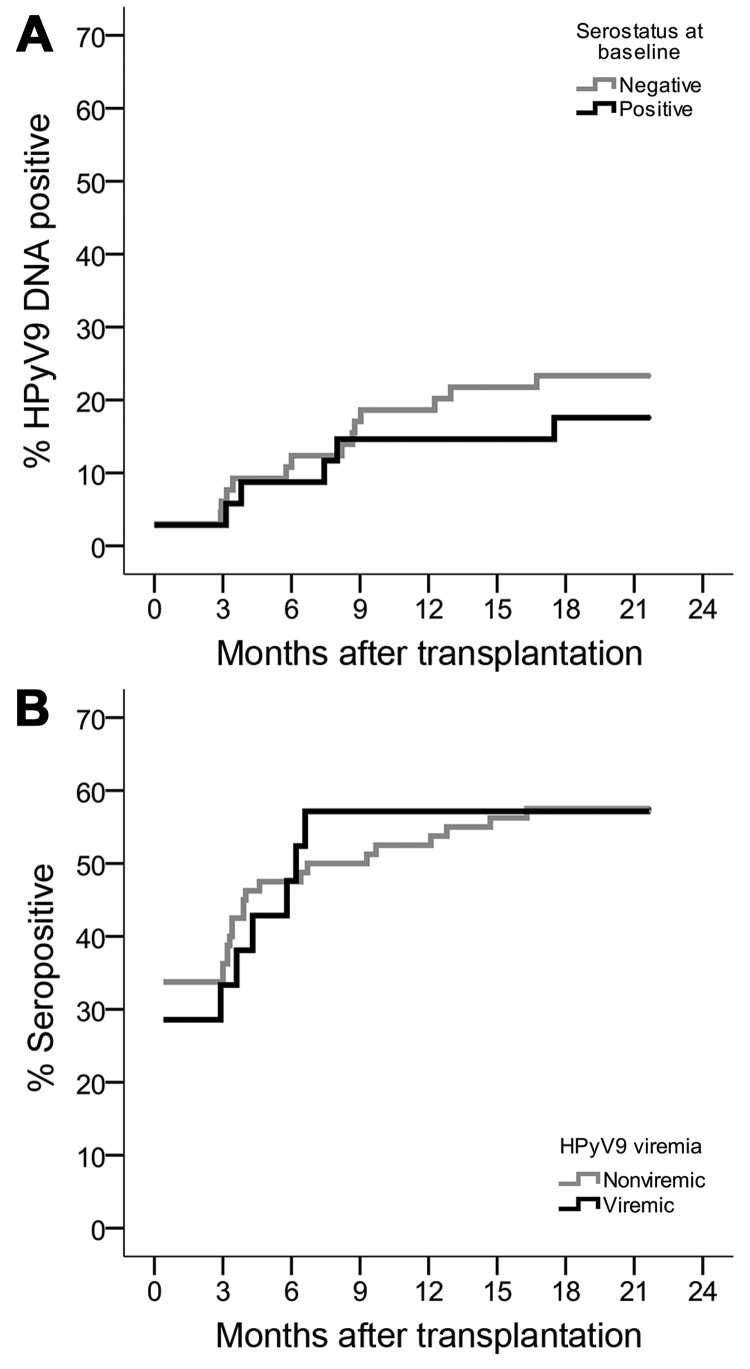
Kaplan-Meier curves showing proportional increase of human polyomavirus 9 (HPyV9) DNA–positive and seropositive transplant patients during 12-month follow-up, the Netherlands. A) Cumulative HPyV9 DNA positivity (viremia) for transplant patients who were seronegative (gray) or seropositive (black) at baseline. B) Cumulative HPyV9 seropositivity for transplant patients who were nonviremic (gray) or viremic (black) at baseline.

Because HPyV9 DNA detection and seroresponses increased after transplantation, we investigated the correlation between these parameters. Comparable proportions of patients who were HPyV9-seropositive and -seronegative at baseline became HPyV9 DNA-positive during follow-up (6/35 [17%] and 15/66 [23%], respectively; p = 0.510 by χ^2^ test) (Figure 3, panel A), and the measured mean viral loads were comparable for the 2 groups (169 and 152 copies/mL, respectively; p = 0.798 by Student *t* test). Furthermore, we analyzed whether the presence of HPyV9 DNA influenced HPyV9 seroreactivity during follow up and found no association (Figure 3, panel B); we also found no association when we compared HPyV9 DNA positivity between high and low seroresponders (above and below median MFI) and seroconverters (data not shown). Stratified analyses for kidney–pancreas and kidney transplant patients did not alter the lack of association. HPyV9 DNA and seroreactivity profiles for patients who seroconverted and/or became viremic during follow-up are shown in the online Technical Appendix.

### Correlation between HPyV9 and BKPyV Viremia

**Figure 4:**
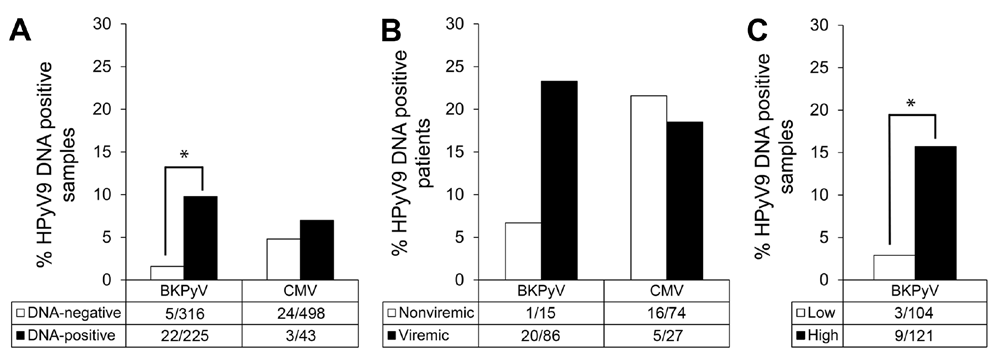
Association between human polyomavirus 9 (HPyV9), BK polyomavirus (BKPyV), and cytomegalovirus (CMV) infection among transplant patients, the Netherlands. A) Percentage of HPyV9 DNA–positive samples among samples that tested negative (white bars) or positive (black bars) for BKPyV and CMV DNA; B) percentage of HPyV9 viremic patients among BKPyV- and CMV-nonviremic (white bars) and viremic (gray bars) patients; C) percentage of HPyV9 DNA–positive samples by measured BKPyV load within the same sample: low, <10^3^ copies/mL (white bars) or high, >10^3^ copies/mL (black bars). Values below bars indicate no. persons positive/total no. tested. *Significant (p<0.05 by χ^2^ test).

Of 541 samples tested, 225 (42%) were BKPyV DNA positive; these samples came from 86 (85%) of the 101 transplant patients. HPyV9 DNA was detected significantly more frequently in BKPyV DNA–positive samples than in BKPyV DNA–negative samples (9.8% vs. 1.6%, respectively; p<0.001 by χ^2^ test) (Figure 4, panel A). During follow-up, HPyV9 DNA was more often detected in BKPyV DNA–positive patients than in BKPyV-negative patients (23.3% vs. 6.7%%, respectively; p = 0.185 by Fisher exact test) (Figure 4, panel B). Furthermore, we divided BKPyV viremic patients into 2 groups, those with high (>10^3^ copies/mL) and low (<10^3^ copies/mL) BKPyV DNA loads, and found HPyV9 DNA–positive patients were overrepresented among patients with high BKPyV loads (p = 0.001 by Fisher exact test; Figure 4, panel C). For 11 (55%) of 20 co-infected patients, BKPyV viremia coincided with HPyV9 viremia; for 8 (40%), BKPyV viremia preceded HPyV9 viremia.

We additionally assessed the presence of viremia caused by CMV, a herpes virus that is not phylogenetically related to HPyV9 and not particularly related to urinary tract infections but that frequently reactivates during immunosuppressive drug use after transplantation. CMV DNA was detected at some point after transplantation in 27 (27%) of the 101 patients (43 [8%] of 541 tested samples). The proportion of HPyV9 DNA–positive samples was similar among CMV DNA positive and negative samples (7% and 5%, respectively; Figure 4, panel A), and no associations were found when comparing HPyV9 DNA positivity and seropositivity among patients who were negative or positive for CMV DNA (Figure 4, panel B; data not shown).

## Discussion

We systematically assessed the presence of HPyV9 DNA and IgG responses in posttransplantation serum samples from kidney transplant patients. These markers of viremia and seroreactivity were shown to increase after transplantation, indicative of active HPyV9 infection, whereas the levels remained stable in matched healthy blood donors.

HPyV9 viremia was detected in 21% of transplant patients at some point within 18 months after transplantation. Most patients were viremic at a single time point, predominantly 3 months after transplantation. The highest mean viral loads were observed immediately after transplantation and decreased gradually over time, but overall HPyV9 loads were low (25–530 copies/mL). Repeat analysis of the complete sample set of 541 serum samples reconfirmed HPyV9 viremia in the same patients (data not shown). At the same time, reanalysis of our cohort showed that the time of a positive finding sometimes differed within viremic patients, compatible with the idea that the viral loads are generally low in persons with HPyV9 viremia and sometimes fall below the PCR detection limit.

Since the identification of HPyV9 in 2011 (*17*), one study has reported detection of the virus in blood from 2% of immunosuppressed patients (*31*), whereas other studies did not find HPyV9 (*32*,*33*). These studies did not report the time of sampling in relation to transplantation and immunosuppression. Our data suggest that active HPyV9 infection is particularly found in the first year after transplantation. After 18 months, only 2.5% of our transplant patients were HPyV9 DNA–positive, with a mean viral load of 51 copies/mL. The use of different primer sets (and probes) in different studies, with different specificity and sensitivity for the detection of HPyV9 DNA, hampers an accurate comparison among studies.

We observed a peak in HPyV9 DNA detection and load in the first 3 months after transplantation, which coincides with the highest dose of immunosuppressive medication administered to these patients. HPyV9 DNA was not detected in serum samples from patients before transplantation or in serum samples from healthy blood donors. Taken together, these observations indicate a close relationship between active HPyV9 infection and transplantation and/or immunosuppression. The higher mean viral load detected in patients who received a combined kidney–pancreas transplant might be the result of the more intensified immunosuppressive regime applied to these patients. Alternatively, the underlying cause of kidney failure might predispose patients for more frequent HPyV9 infection: 94% of kidney–pancreas transplant patients had diabetes, compared with only 6% of kidney transplant patients.

During follow-up, HPyV9 seroprevalence significantly increased among transplant patients, from 33% to 46%, but remained stable at ≈30% in a control group of healthy blood donors among whom no HPyV9 DNA was detected. A previous cross-sectional study observed a comparable difference in HPyV9 seroprevalence between kidney transplant patients (65%) and healthy persons (45%) (*25*).

The detection of HPyV9 DNA and the increase in HPyV9 seroreactivity observed after transplantation could reflect primary infection and reactivation. Polyomavirus infections after transplantation and immunosuppression could result from endogenous reactivation, but proof of this concept is lacking. Infection/reactivation originating from the transplanted organ, as suggested for BKPyV (*34*), should be considered in the case of HPyV9, especially because HPyV9 viremia and baseline HPyV9 seroreactivity were not correlated in this study. HPyV9 viremia was frequently detected in baseline samples from seronegative transplant patients, which suggests donor-derived infection rather than endogenous reactivation. 

Additional analysis of our findings showed that HPyV9 viremia was more prevalent in BKPyV DNA–positive samples and in BKPyV-viremic patients than in their BKPyV DNA–negative equivalents; this association reached statistical significance in BKPyV DNA–positive samples. Stratified analysis revealed that HPyV9 DNA positivity was correlated with high BKPyV load. Taken together, these observations suggest that these related viruses benefit from a joint risk factor present in immunosuppressed kidney transplant patients. The observation that CMV and HPyV9 viremia were not associated, however, suggests that the joint risk factor for the polyomaviruses is not simply explained by immunosuppression, which is a well-known risk factor for CMV.

Although we provide strong evidence for emergence of HPyV9 infection in kidney transplant patients and for association between HPyV9 and BKPyV infection, this study has its limitations. The cohort of kidney transplantation patients we tested was small (n = 101) and was formed >10 years ago. Confirmation of our observations in a more recent and larger cohort will strengthen our findings. Because we were not able to investigate whether donor HPyV9 serostatus correlated with HPyV9 viremia in the recipient, future research might explore the possibility of the donor organ as the source of HPyV9 infection. Furthermore, studies that include urine samples that were not available for our analyses could investigate urinary excretion of HPyV9 in the infected patients and might confirm the epidemiologic correlations we found in serum samples. Finally, the sensitivity of detecting HPyV9 viremic episodes was limited by the 3-month sampling interval. Future studies using a shorter sampling interval would increase the number of measurements.

In conclusion, we identified HPyV9 as an emerging infection in immunosuppressed kidney transplant patients. The observed prevalence of HPyV9 DNA in serum samples (21%) considerably exceeded detection rates of HPyV9 found by others in skin samples of immunocompetent (0.9%) and immuncompromised (2.0%) persons (*35*), which suggests that HPyV9 causes systemic rather than skin infection. Whether HPyV9 is pathogenic in immunocompromised patients, alone or in concert with the well-known pathogen BKPyV, deserves further study. In this context, it would be worthwhile to investigate the course of BKPyV viremia and development of BK virus–associated nephropathy in HPyV9-positive and -negative patients.

Technical Appendix Human polyomavirus 9 seroconversion, viremia, and seroreactivity in transplant patients, the Netherlands.
