# Supplementary material for: Human Polyomavirus 9 Infection in Kidney Transplant Patients
Source: Emerg Infect Dis. 2014 Jun;20(6):991–9. doi: 10.3201/eid2006.140055 (PMC4036759; doi:10.3201/eid2006.140055)
Supplement: Technical Appendix — Human polyomavirus 9 seroconversion, viremia, and seroreactivity in transplant patients, the Netherlands. [file 14-0055-Techapp-s1.pdf]

Article DOI: http://dx.doi.org/10.3201/eid2006.140055

Human Polyomavirus 9 Infection in Kidney Transplant Patients

Technical Appendix

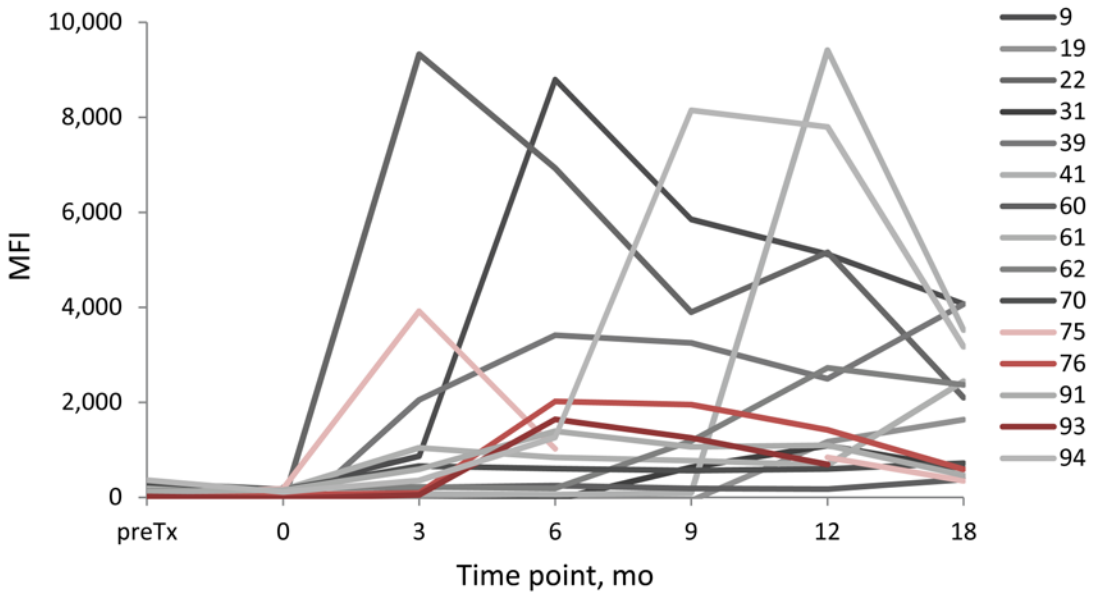

Technical Appendix Figure 1. Human polyomavirus 9 (HPyV9) seroconversion in 15 kidney transplant patients. HPyV9 seroreactivity during follow-up seroconverting transplant patients is shown as measured median fluorescence intensity (MFI) of HPyV9 nonviremic (gray) and viremic (red) patients.

Technical Appendix Figure 2 (following page). Human polyomavirus 9 (HPyV9) viremia and seroreactivity profiles for 21 HPyV9 viremic kidney transplant patients. Individual profiles are given with respect to seroreactivity (shown as MFI, black line) and viremia (shown as measured load in copies/mL [c/ml], bars) at different time points of HPyV9 DNA–positive patients. MFI, median fluoresence intensity.

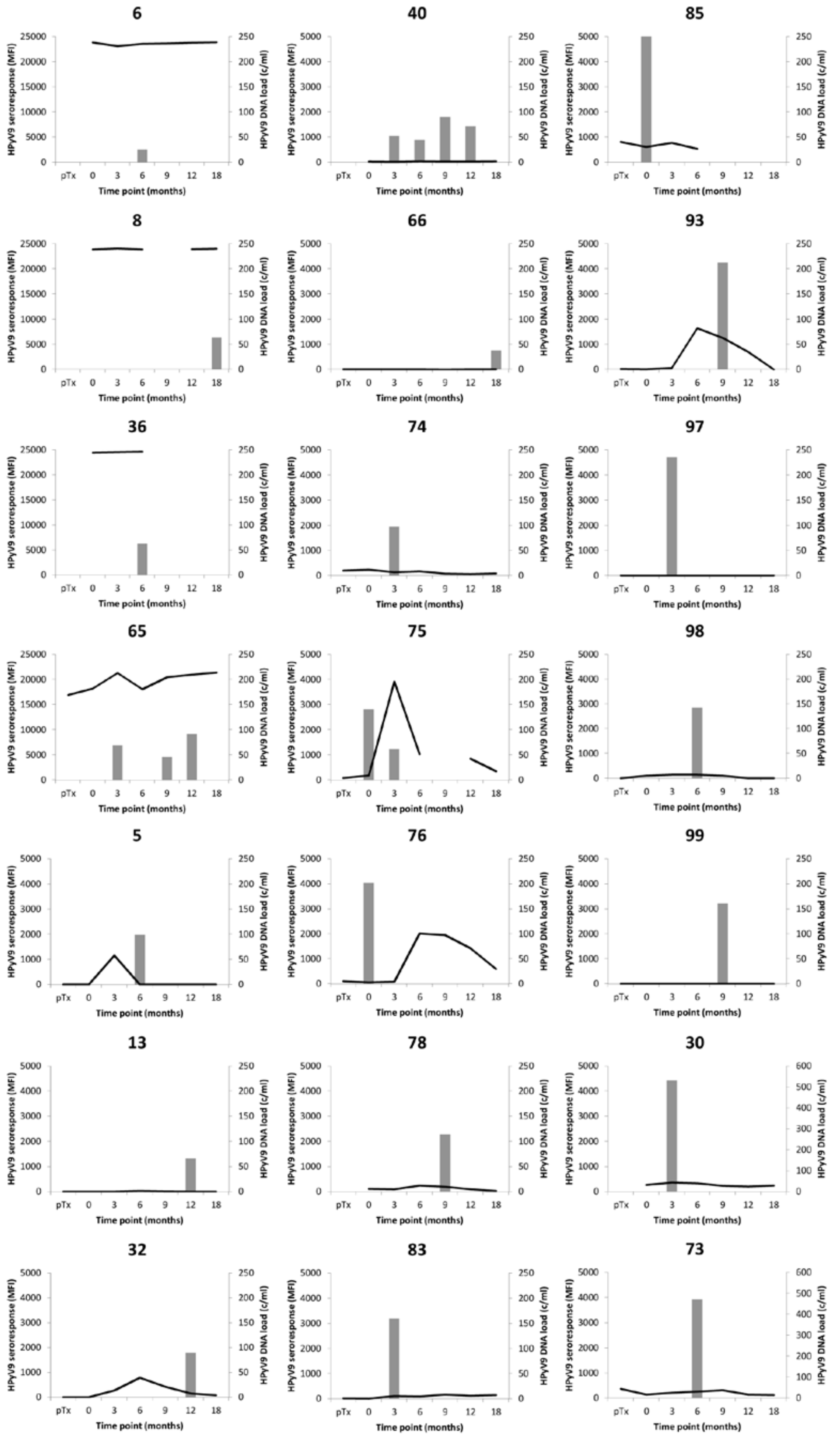
6
40
85
8
66
93
36
74
97
65
75
98
5
76
99
13
78
30
32
83
73
HPyV9 seroresponse (MFI)
HPyV9 DNA load (c/ml)
Time point (months)
pTx
0
3
6
9
12
18
